# *In Vitro* and *In Vivo* Activity of a Novel Locked Nucleic Acid (LNA)-Inhibitor-miR-221 against Multiple Myeloma Cells

**DOI:** 10.1371/journal.pone.0089659

**Published:** 2014-02-21

**Authors:** Maria Teresa Di Martino, Annamaria Gullà, Maria Eugenia Gallo Cantafio, Emanuela Altomare, Nicola Amodio, Emanuela Leone, Eugenio Morelli, Santo Giovanni Lio, Daniele Caracciolo, Marco Rossi, Niels M. Frandsen, Pierosandro Tagliaferri, Pierfrancesco Tassone

**Affiliations:** 1 Department of Experimental and Clinical Medicine, *Magna Graecia* University and Medical Oncology Unit, *Salvatore Venuta* University Campus, Catanzaro, Italy; 2 *T. Campanella* Cancer Center, *Salvatore Venuta* University Campus, Catanzaro, Italy; 3 Pathology Unit, “Giovanni Paolo II” Hospital, Lamezia Terme, Catanzaro, Italy; 4 Exiqon A/S, Vedbaek, Denmark; 5 Sbarro Institute for Cancer Research and Molecular Medicine, Center for Biotechnology, College of Science and Technology, Temple University, Philadelphia, Pennsylvania, United States of America; Istituto dei tumori Fondazione Pascale, Italy

## Abstract

**Background & Aim:**

The miR-221/222 cluster is upregulated in malignant plasma cells from multiple myeloma (MM) patients harboring the t(4;14) translocation. We previously reported that silencing of miR-221/222 by an antisense oligonucleotide induces anti-MM activity and upregulates canonical miR-221/222 targets. The *in vivo* anti-tumor activity occurred when miR-221/222 inhibitors were delivered directly into MM xenografts. The aim of the present study was to evaluate the anti-MM activity of a novel phosphorothioate modified backbone 13-mer locked nucleic acid (LNA)-Inhibitor-miR-221 (LNA-i-miR-221) specifically designed for systemic delivery.

**Methods:**

*In vitro* anti-MM activity of LNA-i-miR-221 was evaluated by cell proliferation and BrdU uptake assays. *In vivo* studies were performed with non-obese diabetic/severe combined immunodeficient (NOD.SCID) mice bearing t(4;14) MM xenografts, which were intraperitoneally or intravenously treated with naked LNA-i-miR-221. RNA extracts from retrieved tumors were analyzed for miR-221 levels and modulation of canonical targets expression. H&E staining and immunohistochemistry were performed on retrieved tumors and mouse vital organs.

**Results:**

*In vitro,* LNA-i-miR-221 exerted strong antagonistic activity against miR-221 and induced upregulation of the endogenous target p27Kip1. It had a marked anti-proliferative effect on t(4;14)-translocated MM cells but not on MM cells not carrying the translocation and not overexpressing miR-221. *In vivo,* systemic treatment with LNA-i-miR-221 triggered significant anti-tumor activity against t(4;14) MM xenografts; it also induced miR-221 downregulation, upregulated p27Kip1 and reduced Ki-67. No behavioral changes or organ-related toxicity were observed in mice as a consequence of treatments.

**Conclusions:**

LNA-i-miR-221 is a highly stable, effective agent against t(4;14) MM cells, and is suitable for systemic use. These data provide the rationale for the clinical development of LNA-i-miR-221 for the treatment of MM.

## Introduction

MicroRNAs (miRNAs) are short non-coding RNAs that are highly deregulated in multiple myeloma (MM) cells [1]–[3]. Recently, a variety of miRNA-profiling studies associated miRNA expression with MM pathogenesis and/or specific molecular sub-entities characterized by chromosomal aberrations and/or gene expression-based risk groups [3]–[5]. More recently, a large body of evidence led to the novel concept that miRNAs may also be tools for the treatment of MM [6]–[11]. Indeed, miR-34a [12] and miR-29b [13], [14] mimics as well as miR-221/222 [15] and miR-21 [16] inhibitors were found to be promising anti-MM therapeutic agents when delivered *in vitro* and *in vivo.*

Among these miRNAs, the miR-221/222 cluster is of particular interest for translational approaches in MM. In fact, miR-221 and 222 are well recognized oncogenic miRNAs that are upregulated in several malignancies [15], [17]–[25]. These miRNAs regulate the expression of a variety of tumor suppressor genes, including the cyclin-dependent kinase inhibitors p27Kip1 and p57Kip2, the pro-apoptotic factors PUMA [19], BIM (BCL2L11) and APAF-1 [26], the key regulator of cell growth and apoptosis PTEN and a metallopeptidase inhibitor TIMP3 [27].

We previously demonstrated that silencing of miR-221/222 with an antisense oligonucleotide (ASO) inhibits proliferation of t(4;14) MM cells *in vitro* and significantly slows the tumor growth in xenografted non-obese diabetic/severe combined immunodeficient (NOD.SCID) mice [15]. We also demonstrated that silencing of miR-221 resulted in higher anti-tumor activity as compared to miR-222, when inhibitors were injected directly into the tumors. Given these promising findings, the aim of the present study was to obtain miR-221 silencing *in vivo* by systemic delivery in order to evaluate the therapeutic potential of this approach in a translational setting.

To obtain an ASO with the properties and stability suitable for systemic delivery, we designed a novel novel phosphorothioate (PS) modified backbone 13-mer locked nucleic acid (LNA)-Inhibitor-miR-221 (LNA-i-miR-221). The LNA/PS technology endows oligonucleotides with unique properties in terms of extreme resistance to enzymatic degradation and improved tissue distribution and pharmacokinetics [28]. In recent years, important and sometimes surprising miRNA functions have been observed after the systemic administration of short highly potent LNA oligonucleotides with a PS backbone. Importantly, these findings were not limited to organs that accumulate large amounts of oligonucleotides, such as the liver [29], [30] or kidney [31], [32]. In fact, efficient silencing of miRNAs has also been reported in a broad range of organs and tissues, such as the lung [33], aorta [34], [35], spleen [36], and even heart [37]–[39], where significant antisense effects have been hard to achieve with other technologies. Of particular relevance to our translational aim are the encouraging results of a limited Phase-2 trial for treatment of HCV infections with a miR-122 inhibitor [40]. That study demonstrated a drug-like property of LNA oligonucleotides together with low systemic toxicity in human healthy subjects carrying HCV infection [40].

In this scenario, we investigated the anti-tumor potential of a novel modified LNA/PS 13-mer LNA-i-miR-221 against t(4;14) MM cells *in vitro* and *in vivo* xenografts. We also evaluated the specificity of anti-miRNA activity on endogenous miRNA-221 targets in these experimental models.

## Materials and Methods

### MM Cells

NCI-H929, OPM2, RPMI-8226, KMS12-BM (available within our research network) [12], [41] and INA-6 cells were cultured in RPMI-1640 medium (Gibco®,Life Technologies, Carlsbad, CA, USA) supplemented with 10% fetal bovine serum, 100 U/ml penicillin, and 100 mg/ml streptomycin (Gibco®) at 37°C in a 5% CO_2_ atmosphere. The IL-6 dependent MM cell line INA-6 (kindly provided by Dr. Renate Burger, University of Erlangen-Nuernberg, Erlangen, Germany) was cultured with rhIL-6 (R&D Systems, Minneapolis, MN) as previously reported [42]–[45].

### Design and Synthesis of LNA Oligonucleotides

Custom LNA oligonucleotides were provided by Exiqon (Vedbaek, Denmark). LNA-i-miR-221 is a 13-mer DNA/LNA oligonucleotide whose sequence is CAGACAATGTAGC. A mismatch LNA 13-mer oligonucleotide (LNA-i-miR-NC) with the same sequence (except that bases were swapped at four positions: CTGAGAAAGTACC), LNA residues and melting temperature served as control. Both oligonucleotides had a fully PS-modified backbone. They were purified by HPLC followed by Na+ salt exchange and lyophilization.

### Cell Proliferation Assays

For cell growth analysis, we used the Neon® Transfection System (Life Technologies) to transfect MM cells with LNA-i-miR-221 and LNA-i-miR-NC, or with miR-221/222 mimics (Life Technologies), as previously described [15]. 2×10^5^ MM cells were electroporated with LNA-i-miR-221 or scrambled control, seeded in 96-well plates and tested with the Cell Counting Kit-8 colorimetric assay (CCK-8, Dojindo Molecular Technologies, Japan). Absorbances were measured (450 nm) at different time points with the GloMax-multi detection system (Promega, Madison, WI, USA). To determine BrdU uptake, we measured the incorporation of BrdU into DNA strands using the DELFIA cell proliferation kit (Perkin-Elmer, Waltham, MA, USA). After transfection with LNA-i-miR-221 or scrambled control, 1×10^4^ cells were seeded in 96-well plates. Then, BrdU (10 µM) was added at 24-hour intervals and incorporation was measured by time-resolved fluorescence of a europium-chelate on a Wallac Victor 2 multilabel counter (Perkin-Elmer). All assays were repeated at least twice.

### Quantitative Real-time Amplification (q-RT-PCR) of miRNAs and mRNAs

Total RNA (tRNA) was isolated from MM cells cultured *in vitro* and also from normal (liver, kidneys and heart) mouse tissue, or from tumor xenografts by TRIzol® Reagent (Invitrogen, Life Technologies). Tissue disruption was performed using the TissueRuptor® system (Qiagen, Venlo, Netherlands). We used single-tube TaqMan miRNA assays (Applied Biosystems, Life Technologies, Carlsbad, CA, USA) to detect and quantify mature miR-221 (assay ID 000524) using the ViiA7 detection system (Applied Biosystems). For the analysis of xenograft and vital mouse tissue, miRNA expression was normalized on the RNU44 (assay ID 001094) and snoRNA-202 (assay ID 001232) small housekeeping mRNAs (Applied Biosystems), respectively. To measure p27Kip1 mRNA levels, we obtained an oligo-dT-primed cDNA using the High Capacity cDNA Reverse Transcription Kit (Applied Biosystems), and Taqman assay (assay ID Hs01597588_m1). Normalization was performed with the GAPDH (assay ID Hs03929097_g1, Applied Biosystems) housekeeping gene. Comparative RT-PCR was performed in triplicate, and included no template controls. Relative expression was calculated with the comparative cross threshold (Ct) method [46].

### Western Blotting Analysis

SDS-PAGE and Western blotting were performed according to standard protocols. Briefly, cells were homogenized in lysis buffer containing 15 mM Tris/HCl pH 7.5, 120 mM NaCl, 25 mM KCl, 1 mM EDTA, 0.5% Triton X-100, and Halt Protease Inhibitor Single-Use cocktail (100X, Thermo Scientific, Waltham, MA, USA). Whole cell lysates (50 µg per line) from transfected cell lines were separated using 4–12% Novex Bis-Tris SDS-acrylamide gels (Invitrogen), electro-transferred onto nitrocellulose membranes (Bio-Rad, Hercules, CA, USA), and immunoblotted with the p27Kip1 (SX53G8.5) mouse mAb (Cell Signaling, Beverly, MA, USA). Membranes were washed 3 times in PBS-Tween and then incubated with a secondary antibody conjugated with horseradish peroxidase in 0.5% milk for 2 hours at room temperature. Chemiluminescence was detected using Western Blotting Luminol Reagent (sc-2048, Santa Cruz, Dallas, TX, USA). Signal intensity was quantified with the Quantity One Analyzing System (Bio-Rad).

### Luciferase Reporter Experiments

The 3′UTR sequence of the CDKN1B (p27Kip1) gene was purchased from OriGene Technologies (Rockville, MD, USA). NCI-H929 cells were co-transfected by electroporation, as described above, with 5 µg of the firefly luciferase reporter vector, 0.5 µg of the control vector containing Renilla luciferase, pRL-TK (Promega) and 100 nM of the LNA-i-miR-221 inhibitor or LNA-i-miR-NC. Firefly and Renilla luciferase activities were measured sequentially using the Dual-Luciferase Reporter Assay (Promega) 24 and 48 hours after transfection, and results were normalized with Renilla luciferase. A pLightSwitch_3′UTR Reporter Vector containing a synthetic target consisting of sequence repeats fully complementary to the miR-221-3p, based on miRBase 16 annotations, was purchased from Switch Gear Genomics (Menlo Park, CA, USA). The miR-221-3p synthetic target was cloned downstream of the Renilla luminescent reporter gene (RenSP). 10 µg of pLightSwitch_3′UTR reporter plasmid and 100 nM of LNA-i-miR-221 inhibitor or scrambled control were used to co-transfect 1×10^6^ MM cells. Twenty-four hours after transfection, 100 µl of LightSwitch Assay reagent (SwitchGear Genomics) were added to each well. Plates were incubated for 30 min at room temperature and the luciferase reporter signal was recorded on a Glomax detection System luminometer (Promega). Each experiment was performed at least three times and each sample was assayed in triplicate.

### Animals and *in vivo* Model of Human MM

Male CB-17 NOD.SCID mice (6- to 8-weeks old; Harlan Laboratories, Inc., Indianapolis, IN, USA) were housed and monitored in University Magna Graecia Animal Research Facility. The rational design, the use of mice and the experimental procedures within the AIRC Special Program Molecular Clinical Oncology – “5 per mille”, grant n. 9980, 2010–15, were approved by the Ethical Committee of the University/Hospital (Azienda Policlinico Materdomini) of Magna Graecia, Catanzaro, Italy (Approval: 20.12.2010). Experimental protocols (n. 235/30.6.2011) were also approved by the National Directorate of Veterinary Services (Italy). Tumor sizes were measured as previously described [12]. When tumors measured 2 cm^3^, or in the event of paralysis or if mice quality of life appeared to be seriously impaired, animals were sacrified by CO_2_ inhalation previous chloralium hydrate anesthesia (400 mg/kg), to prevent unnecessary suffering. For *in vivo* studies we used a mouse model of human MM [47]. To induce MM, 1×10^6^ OPM-2 cells in 100 µL RPMI-1640 medium were subcutaneously injected into the interscapular area of NOD.SCID mice. When tumors became palpable (approximately 10 days after the injection of MM cells) mice were randomized into 2 groups (5 animals per group) and treated with LNA-i-miR-221 or scrambled control. We performed two different experiment. In the first one, animals received an intraperitonal injection of 25 mg/kg of LNA inhibitors per week for two weeks. In a subsequent experiment animals received 2 intravenous injections of 25 mg/kg of LNA inhibitors per week for two weeks, then tumors were retrieved from animals and placed in 10% formalin for histology, in RNA*later*® for RNA isolation or stored at −80°C for protein analysis. Vital organs including liver, kidney and heart were collected for histology or RNA isolation and stored at the conditions reported above.

### Histology and Immunohistochemistry

Tissues were immediately fixed in 4% buffered formaldehyde, and 24 h later, washed, dehydrated and embedded in paraffin. Four-µm thick sections were mounted on poly-lysine slides and stained with hematoxylin-eosin. Samples were examined under an optical OLYMPUS BX51 microscope (Olympus Corporation, Tokyo, Japan).

For immunohistochemistry, 3-µm-thick tumor slices were deparaffinized and treated with Epitope Retrieval Solution 2 (EDTA-buffer pH 8.8) at 98°C for 20 min. After washing, peroxidase was blocked by exposing samples to the Bond Polymer for 10 min. All procedures were performed with the Benchmark XT - Automated Immunohistochemistry instrument (Ventana Medical Systems, Oro Valley, AZ, USA). Tissues were washed again and then incubated with the rabbit monoclonal primary antibody CONFIRM anti-Ki-67 (Ventana, clone 30-9; 1∶150) or p27Kip1 (Ventana, clone SX53G8). Tissues were then incubated with DAB-Chromogen (8 min) and slides were counterstained with hematoxylin (12 min).

### Statistical Analysis

All *in vitro* experiments were repeated at least 3 times and performed in triplicate; a representative experiment was showed in figures ±SD. Statistical significance of the *in vivo* growth inhibition observed in LNA-i-miR-221-treated mice compared with scrambled control group was determined using Student’s *t* test. The minimal level of significance was specified as *P*<0.05. All statistical analyses were determined using GraphPad software (www.graphpad.com). Graphs were obtained using Microsoft Office Excel tool.

## Results

### *In vitro* Silencing of miR-221 by LNA-i-miR-221

**Figure 1:**
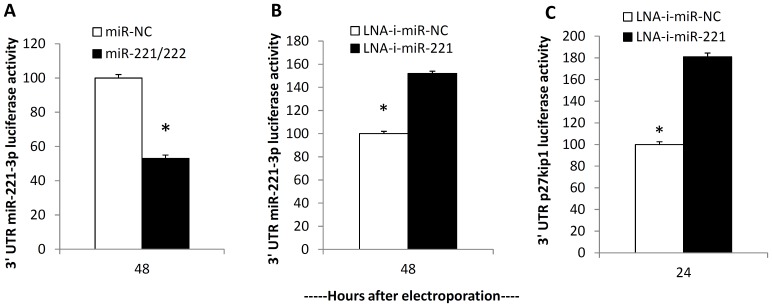
LNA-i-miR-221 specifically recognizes the miR-221 complementary sequence. Luciferase reporter assay of NCI-H929 cells co-transfected with pLightSwitch_3′UTR Reporter Vector containing the miR-221-3p synthetic sequence cloned downstream of the Renilla luminescent reporter gene (RenSP) and miR-221/222 mimics (A) or LNA-i-miR-221 (B) or scrambled sequences as control. The firefly luciferase activity was normalized to Renilla luciferase activity. The data are shown as relative luciferase activity of miR-221/222-transfected cells versus the control (miR-NC) or LNA-i-miR-221-transfected cells versus the scrambled control (LNA-i-miR-NC). C) Dual-luciferase assay of NCI-H929 cells co-transfected with firefly luciferase constructs containing the 3′UTR of p27Kip1 and LNA-i-miR-221 or scrambled oligonucleotides (NC) as indicated. Firefly luciferase activity was normalized to r Renilla luciferase activity. The data are shown as relative luciferase activity of LNA-i-miR-221-transfected cells versus the control (NC).

To evaluate the anti-tumor potential of the novel 13-mer inhibitor, LNA-i-miR-221, we first verified that it effectively knocked down miR-221 function in MM cells cultured *in vitro*. To this aim we used the 3′UTR reporter (luciferase renilla/firefly) construct containing the miR-221 target site. This construct was co-transfected either with miR-221/222 mimics or LNA-i-miR-221 into NCI-H929 cells. As predicted, luciferase activity was reduced when miR-221/222 mimics were co-transfected with 3′UTR reporter plasmid (Fig. 1A) and increased in LNA-i-miR-221-transfected MM cells (Fig. 1B), thereby indicating efficient and stable binding to the miRNA target.

**Figure 2:**
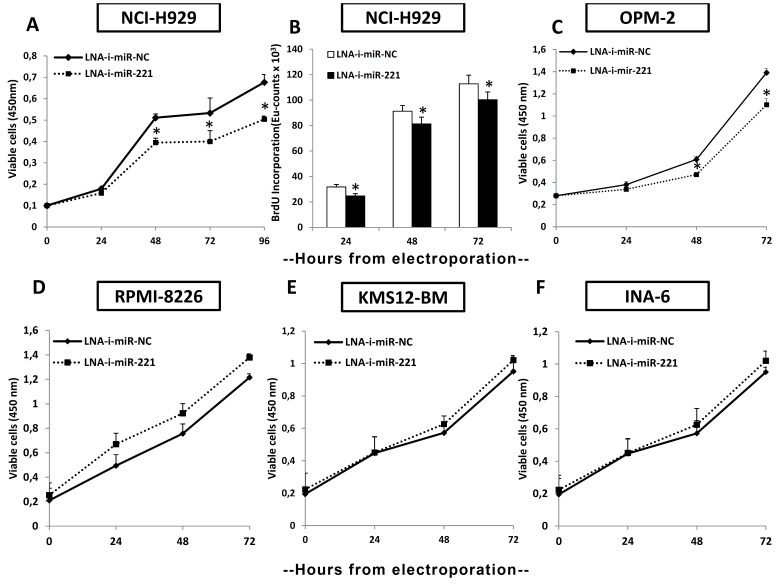
Antiproliferative effects induced by transient expression of LNA-i-miR-221 in MM cell lines. Effects on proliferation (A) and BrdU uptake (B) in NCI-H929 cells induced by transient LNA-miR-221 inhibitor (LNA-i-miR-221) transfection compared to scrambled control (LNA-i-miR-NC). C) OPM-2 cell growth inhibition. MM cells not bearing t(4;14): RPMI-8226 (D), KMS12-BM (E) and INA-6 (F) are not affected by transfection with LNA-i-miR-221 as compared to scrambled LNA sequences. Averaged values ±SD from 3 independent experiments.

To probe further the effect of LNA-i-miR-221 on derepression of p27Kip1, which is a well established miR-221 canonical target, we co-transfected a luciferase reporter vector containing the 3′UTR region of p27Kip1 into MM cells. We found that the luciferase/Renilla ratio was much higher in LNA-i-miR-221 than in the control transfected cells (Fig. 1C). We next evaluated the effect of LNA-i-miR-221 on cell proliferation and BrdU incorporation at different time points after transfection of t(4;14) MM cells overexpressing miR-221. As shown in Figure 2A, 48 hours after transfection, cell proliferation was 25% lower in LNA-i-miR-221-transfected NCI-H929 cells than in control cells. The BrdU incorporation assay produced similar results (Fig. 2B). A 20% of cell growth inhibition was also detected in LNA-i-miR-221-transfected t(4;14) OPM-2 cells (Fig. 2C).

Subsequently, we evaluated the inhibitory activity of LNA-i-miR-221 on MM cells not bearing the t(4;14) translocation and expressing low levels of miR-221 [15]. No significant proliferation changes were observed in these cells 24, 48 and 72 hours after transfection (Fig. 2D, E and F). This finding demonstrates that, as expected, the inhibition of cell growth was related to the miR-221 overexpression.

**Figure 3:**
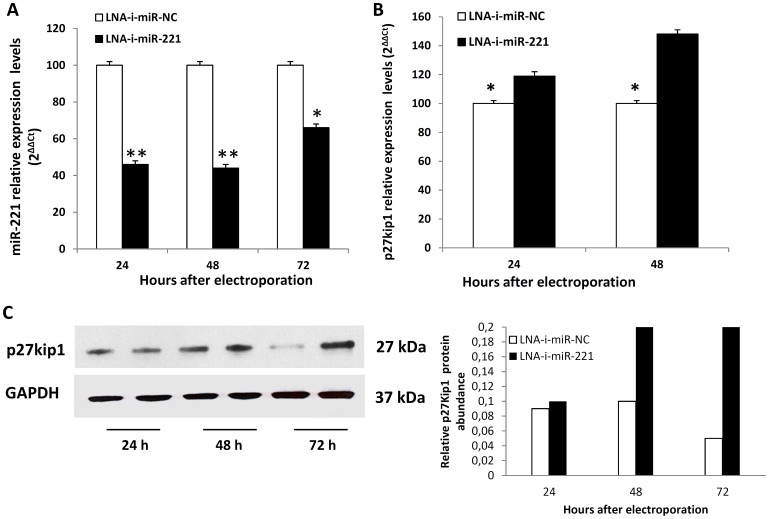
Molecular effects induced by LNA-i-miR-221 transfection in MM cells. miR-221(A) q-RT-PCR 24, 48 and 72 hours after transfection with LNA-i-miR-221 and LNA-i-miR-NC in NCI-H929 cells. The results are shown as miRNA expression levels after normalization with RNU44 and ΔΔCt calculations. Data represent the average of 3 independent experiments ±SD. B) q-RT-PCR of p27Kip1 mRNA expression 24 and 48 hours after transfection with LNA-i-miR-221 or scrambled control in NCI-H929 cells. Data represent the average of 3 independent experiments ±SD after normalization with GAPDH mRNA and ΔΔCt calculations. (*) P<0.05, (**) P<0.01. C) Western blot analysis of p27Kip1 protein in NCI-H929 cells 24, 48 and 72 hours after transfection with LNA-i-miR-221 or control. GAPDH was used as protein loading control.

The q-RT-PCR evaluation of miR-221 in NCI-H929 cells showed that miR-221 levels were 50% lower 24 and 48 hours after transfection, and 35% lower 72 hours after transfection compared with the control (Fig. 3A and B). Down-regulation of miR-221 was associated with up-regulation of p27Kip1 at both mRNA and protein level (Fig. 3C and D, respectively).

### *In vivo* Activity of LNA-i-miR-221 Inhibitor by Systemic Injection

**Figure 4:**
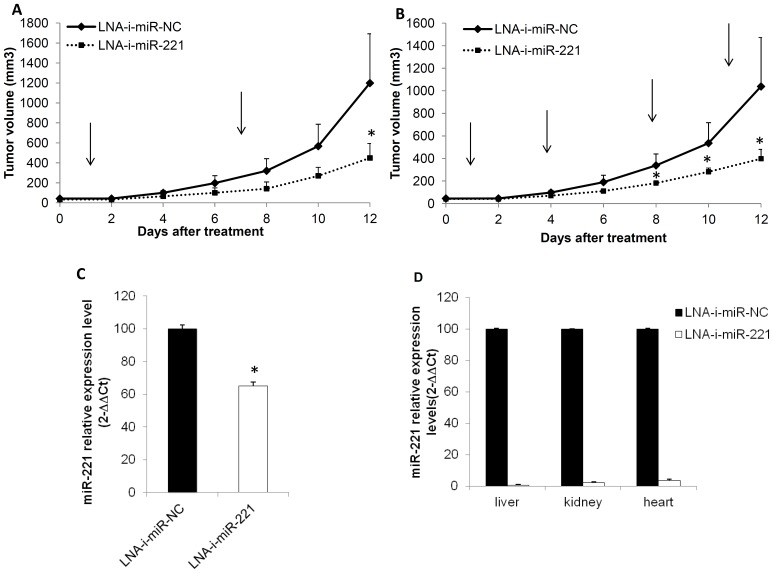
*In vivo* anti-tumor activity of LNA-i-miR-221 in MM-xenografted SCID/NOD mice. Effects of LNA-i-miR-221 after 2 intraperitoneal injections of 25 mg/kg (A) and 4 intravenous injections of 25 mg/kg (B). MM-xenografted mice were treated with saline solution of LNA-i-miR-221 or LNA-i-miR-NC as control. The average tumor volume of each group ±SD is reported. P values were calculated for miRNA inhibitors *versus* scrambled oligonucleotides (NC) and significant P values (*P*<0.05) are indicated by asterisks. C) q-RT-PCR of miR-221 in retrieved tumors treated intravenously with LNA-i-miR-221 or LNA-i-miR-NC. The results are shown as averaged miRNA expression after normalization with RNU44 and ΔΔCt calculations as compared to control (miRNA expression in LNA-i-miR-NC treated animals) ±SD.*P<0.05. D) q-RT-PCR of miR-221 in liver, kidney and heart tissue biopsies of mice after 2 weeks of treatment. The results are shown for each organ as average miRNA expression after normalization with snoRNA-202 and ΔΔCt calculations with respect to miRNA expression in LNA-i-miR-NC-treated animals. The results represents the average value obtained in 3 different mice ±SD. **P<0.01.

To assess the translational relevance of our findings, we evaluated the anti-tumor potential of LNA-i-miR-221 in human MM xenografts in NOD.SCID mice. When t(4;14) MM xenografts became palpable, mice were randomized into 2 groups and treated with LNA-i-miR-221 or scrambled control by injection of naked (unformulated) inhibitors. After the intraperitoneal injection of LNA-i-miR-221 (25 mg/kg once a week for 2 weeks), tumors were significantly inhibited as compared to the control group (*p*<0.5) (Fig. 4A). A similar result was obtained in tumor-bearing mice randomized to receive intravenous injection twice a week for two weeks (Fig. 4B).

### Biological Effects Induced by LNA-i-miR-221 *in vivo*

**Figure 5:**
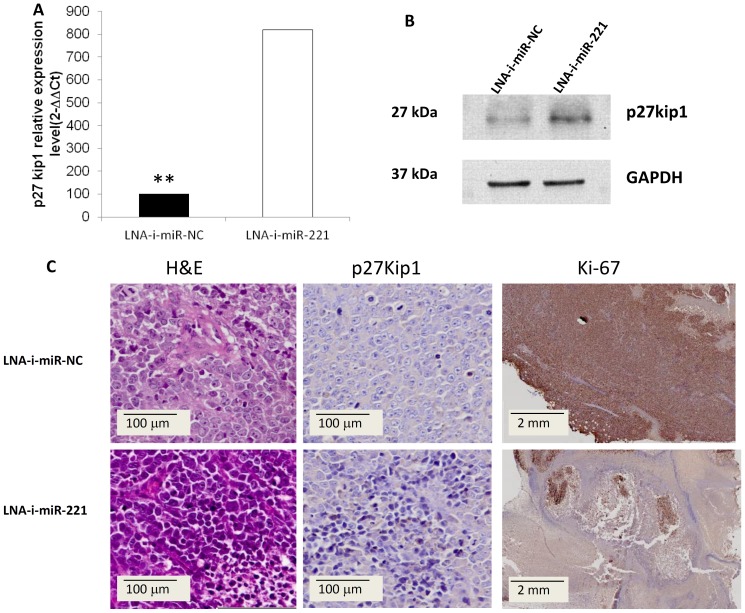
LNA-i-miR-221 antiproliferative activity and target silencing in retrieved MM xenografted tumors. A) q-RT-PCR of p27Kip1 mRNA levels in treated tumors retrieved from mice after intravenous LNA-i-miR-221 treatment. Raw Ct values were normalized to GAPDH housekeeping mRNA and expressed as ΔΔCt values calculated using the comparative cross threshold method (miRNA expression in LNA-i-miR-NC treated animals) ±SD. B) Western blot analysis of p27Kip1 protein in retrieved tumors from mice treated with LNA-i-miR-221 inhibitors or LNA-i-miR-NC. GAPDH was the protein loading control. C) H&E (200-fold magnification), p27Kip1 (200-fold magnification) and Ki-67 (10–fold magnification) immunohistochemistry staining of xenografted tumors retrieved from treated animals. Representative images are shown.

To investigate the molecular effects induced by LNA-i-miR-221 on tumor cells *in vivo,* we measured miR-221 expression levels in retrieved xenografts and in different mouse tissues after intravenous administration of the inhibitor. As shown in Figure 4C, miR-221 levels were 34% lower in LNA-i-miR-221-treated tumors than in controls. We also evaluated p27Kip1 expression at mRNA and protein levels in xenografts retrieved after exposure to LNA-i-miR-221 intravenous treatment and we found that the levels of p27Kip1 mRNA (Fig. 5A) and protein (Fig. 5B) were much higher in tumors from animals treated with LNA-i-miR-221 as compared to controls. Moreover, at immunohistochemistry, p27Kip1 expression was higher in LNA-i-miR-221-treated tumors as compared to controls (60% *versus* 10% of p27Kip1-positive cells, respectively) and the Ki-67 proliferation index was greatly reduced (Fig. 5C). miR-221 was barely detectable in the liver, kidney and heart tissues of treated animals (Fig. 4D).

Exposure to LNA-i-miR-221 did not cause any significant behavioral changes or weight loss in animals. Histology did not reveal any organ damage or pathologic changes in the liver, kidney or heart tissues of treated mice (data not shown), which indicates that the treatment was not toxic.

## Discussion

miR-221 acts as an oncomiR in many human solid and hematologic neoplasms. Overexpression of miR-221 interferes with a wide set of gene targets involved in proliferation and apoptosis in a variety of these malignancies [12], [20], [35]. We previously demonstrated that silencing the miR-221/222 by specific inhibitors in MM cells bearing the t(4;14) translocation resulted in powerful anti-tumor activity *in vitro* and in murine models of human MM [15]. We also demonstrated that miR-221 inhibitors were more active than miR-222 inhibitors. However, these effects were obtained by injecting the inhibitors directly into the tumors. The aim of the present study was to evaluate the activity of a novel 13-mer LNA-i-miR-221 inhibitor specifically designed for systemic delivery.

We speculate that, *in vitro,* this novel LNA-i-miR-221 formed strong heteroduplexes with miR-221 as suggested by reduced miR-221 levels as assessed by quantitative PCR. Moreover, LNA-i-miR-221 significantly impaired miR-221 function as demonstrated by strong derepression of p27Kip1, which is a direct target of miR-221, by functional luciferase assays.

The specificity of miR-221 inhibition in our study is demonstrated by the lack of significant changes in the proliferation rate of MM cells not bearing the t(4;14) translocation and expressing low miR-221 levels. Although the MM cells were hard-to-transfect, t(4;14) cells showed an anti-proliferative effect *in vitro,* which was also observed in our *in vivo* MM model. After two weeks of exposure to LNA-i-miR-221, the tumor growth was significantly inhibited as compared to xenografted control mice. However, we found that systemically administered LNA-i-miR-221 exerted a significant anti-MM activity *in vivo*. The latter effect was associated with reduced miR-221 expression and upregulation of p27Kip1.

Importantly, miRNA inhibition has been reported to be specific *in vivo* using the LNA-modified PS oligonucleotide miR-122 inhibitor as an anti-hepatitis C agent [48]. Moreover, LNA-miR-122 inhibitors has been clinically evaluated in hepatitis C healthy carriers [40], which indicates the clinical feasibility of LNA-based miRNA inhibition. Systemic toxicity was limited in patients treated with the inhibitor and serum HCV levels were steadily knocked down.

Despite these encouraging clinical findings, the development of effective and safe approaches for sequence-specific antagonism of miRNAs *in vivo* remains a scientific and therapeutic challenge. In fact, the development of LNA oligonucleotides is based on the concept that inhibition of target function depends on binding affinity to allow efficient target derepression. The LNA content influences the melting temperature of the duplex, increasing about 2–8°C for each LNA with respect to the corresponding DNA nucleotide duplex. Moreover, combining high-affinity-LNA with PS modifications confers high stability *versus* exo- and endo-nuclease cleavage thereby enabling the systemic delivery of unconjugated, saline-formulated LNA-modified oligonucleotides (LNA-i-miRNA). The LNA-i-miR-221 oligonucleotide that has a complete PS backbone resulted in lasting and effective miRNA antagonism as shown by the decrease of both miRNAs and by the upregulation of p27Kip1 up to 2 weeks after the last LNA-i-miR-221 treatment. These observations are in accordance with the previously reported prolonged LNA-miR-122 antagonism in primates [48]. Importantly, we did not observe any acute or subchronic toxicities in LNA-i-miR-221-treated mice as assessed by animal observation and histopatology evaluation of vital organs. Taken together, these findings indicate that LNA-i-miR-221 is a promising anti-MM agent that warrants investigation in primate toxicology studies. If the safety profile is satisfactory, LNA-i-miR-221 may be considered for early clinical trials in t(4;14) MM patients, and in patients with other malignancies characterized by miR-221 deregulation.
